# Giant periscapular lipoma unmasked by post-bariatric surgery weight loss: a case report

**DOI:** 10.1093/jscr/rjae817

**Published:** 2024-12-27

**Authors:** Hussain Mohammad, Suaad Almajed, Norah Nawaf, Nawar AlMulla, Ali Lari, Ali Jarragh

**Affiliations:** Department of Orthopedic Surgery, AlRazi National Orthopedic Hospital, Kuwait City, Kuwait; Department of Orthopedic Surgery, AlRazi National Orthopedic Hospital, Kuwait City, Kuwait; Department of Orthopedic Surgery, AlRazi National Orthopedic Hospital, Kuwait City, Kuwait; Department of Orthopedic Surgery, AlRazi National Orthopedic Hospital, Kuwait City, Kuwait; Department of Orthopedic Surgery, AlRazi National Orthopedic Hospital, Kuwait City, Kuwait; Department of Surgery, Kuwait University, Kuwait City, Kuwait

**Keywords:** giant lipoma, intramuscular tumour, surgical excision, obesity, weight loss, case report

## Abstract

Giant lipomas, rare benign tumours composed of mature adipose tissue, represent only 1% of all lipomas, typically exceeding 10 cm in diameter or weighing over 1000 g. These tumours can cause nerve compression, discomfort, or functional impairment, necessitating surgical excision. We report a 52-year-old male with a giant intramuscular lipoma in the periscapular region, initially identified following significant weight loss after bariatric surgery. Clinical evaluation revealed a 15 × 20 cm mass, confirmed via computed tomography (CT) scan. Surgical excision was performed, followed by histopathological analysis, which confirmed a benign lipoma. Postoperative management was complicated by seroma formation, requiring drainage. This case underscores the importance of early diagnosis, imaging, and appropriate surgical management for large lipomas to prevent complications and recurrence while ensuring optimal cosmetic outcomes.

## Introduction

Lipomas are noncancerous tumours composed of mature fat cells, with an incidence of 2.1 per 1000 individuals [1]. They are classified into subcutaneous, subfascial, intermuscular, and intramuscular lipomas [2–4]. Giant lipomas, defined as tumours measuring at least 10 cm in diameter or weighing 1000 g, can cause lymphedema, discomfort, or nerve compression due to their size and location [5, 6]. These are rare, representing 1% of all lipomas, and are often found on the neck, shoulders, back, abdomen, arms, or thighs [4, 7]. Surgical excision is the standard treatment, although it can be complicated by the tumour’s size and location. Postoperative histological assessment is essential to confirm the diagnosis [4]. This case report presents a 52-year-old male with a giant lipoma in the periscapular muscles, emphasizing early diagnosis and management.

## Case presentation

A 52-year-old male presented to the orthopaedics clinic with swelling in the right upper back. The patient reported noticing the swelling 3 months earlier after significant weight loss due to bariatric surgery. His medical history included diabetes, hyperlipidaemia, hypertension, hyperuricemia, and irritable bowel syndrome. He was otherwise asymptomatic, with no discomfort related to the swelling.

**Figure 1:**
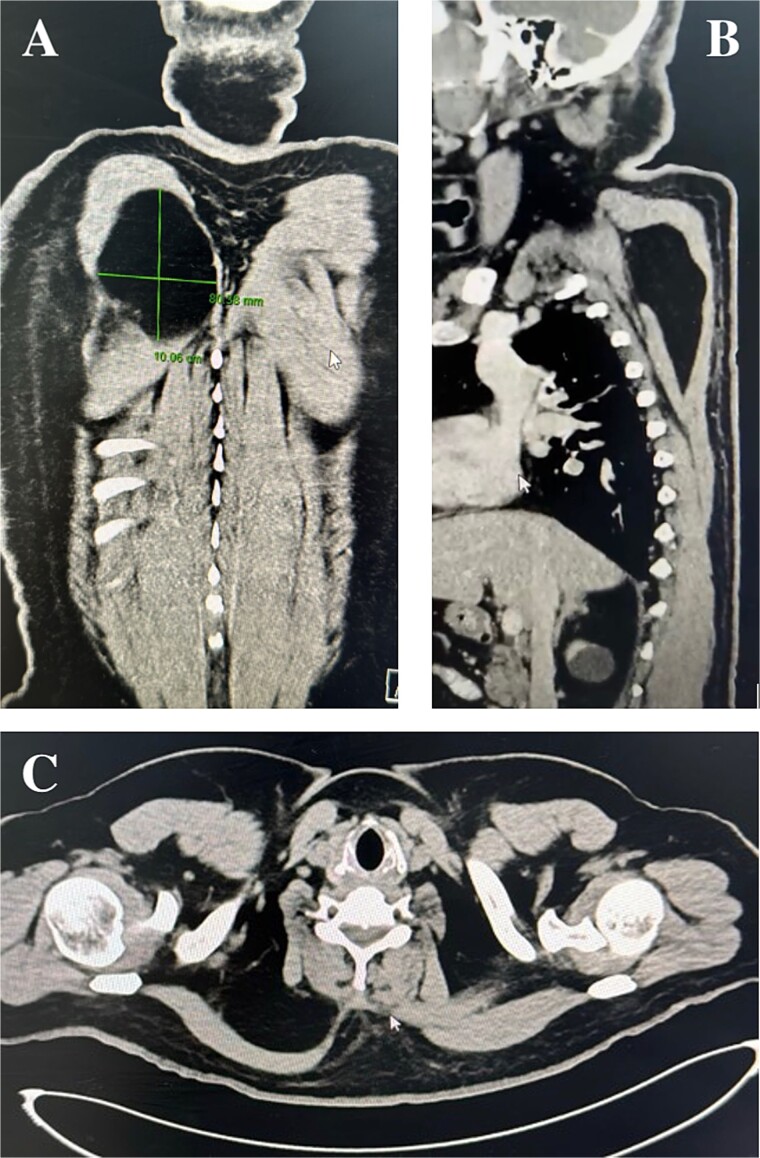
Computed tomography scan: Coronal, sagittal, and axial planes of the lipoma.

**Figure 2:**
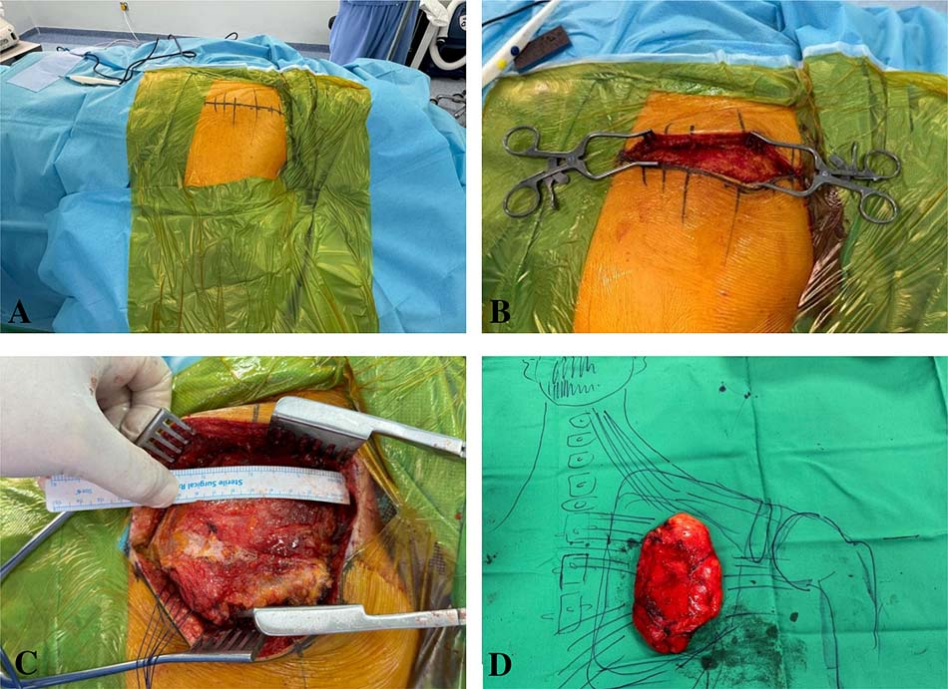
Intraoperative imaging: Marking the incision, exposing the periscapular muscles, dissecting the lipoma, and displaying it over a drawn figure of the anatomy.

Physical examination identified a well-circumscribed mass measuring 15 cm × 20 cm in the right scapular region. A computed tomography (CT) scan showed a low-attenuation intramuscular mass without calcification (Fig. 1A-C). A magnetic resonant imaging (MRI) scan was recommended as the primary investigation, but the patient refused. Surgical excision was performed with the patient in the prone position (Fig. 2A). A 15 cm incision was made between the spinal process and the scapula’s medial border (Fig. 2B). Subcutaneous flaps were raised, revealing the palpable mass beneath the trapezius and rhomboid muscles. The mass, appearing benign, was removed and sent for histopathological analysis (Fig. 2C and D). The muscle fascia was repaired, and the incision was closed in layers over a surgical drain. The procedure was uneventful, and the patient was transferred back to the ward.

**Figure 3:**
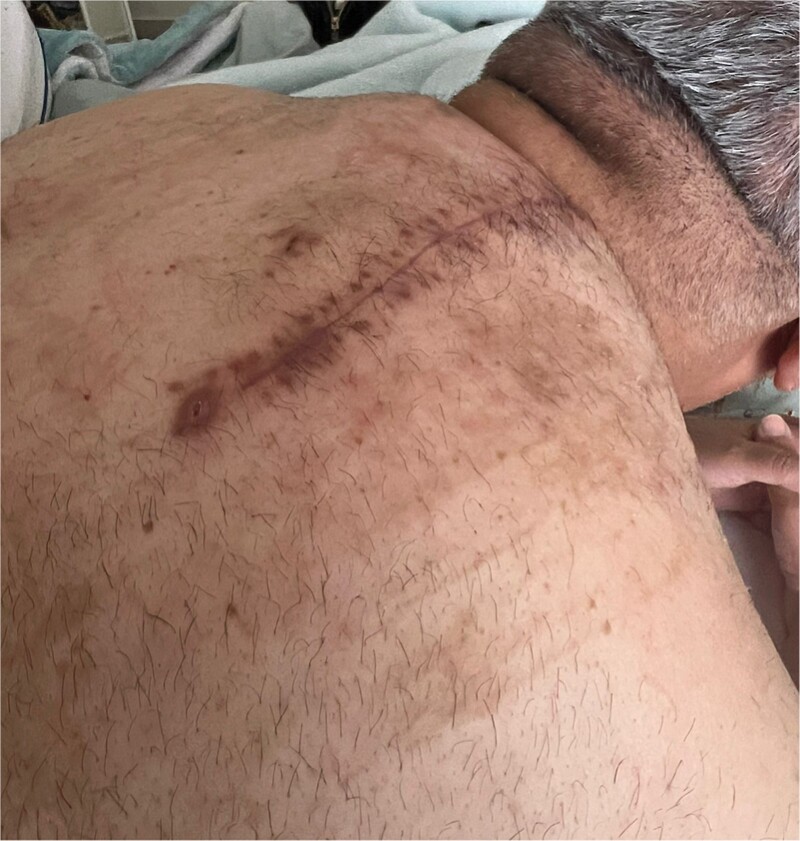
Postoperative imaging: Fully healed surgical incision.

**Figure 4:**
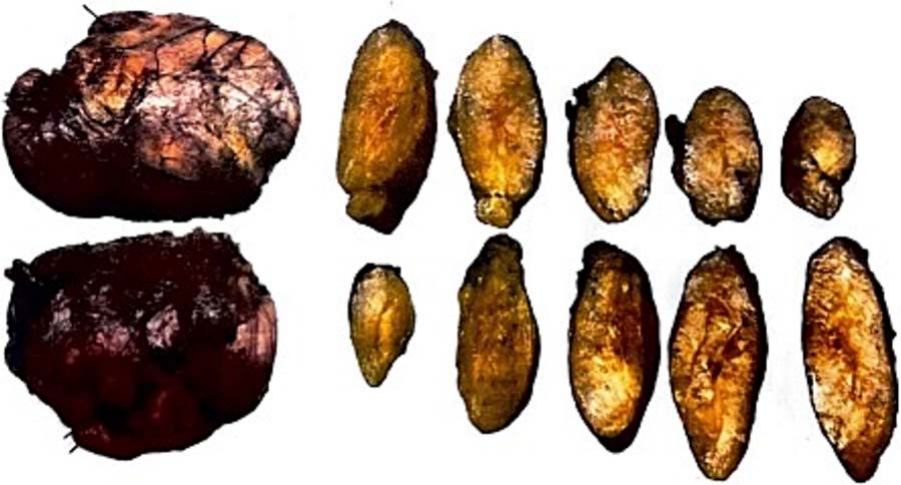
Pathological assessment: Gross images of the specimen before and after dissection.

Postoperatively, the patient developed a seroma, which required evacuation and drain placement until resolution (Fig. 3). The pathology report described the mass as a thinly encapsulated, oval-shaped fatty lump weighing 200 g and measuring 12 × 9.3 × 3 cm. The cut surface showed soft yellow fatty tissue without cyst formation, haemorrhage, or necrosis (Fig. 4). Microscopic analysis confirmed a benign lipoma consisting of mature fibroadipose tissue.

## Discussion

Lipomas originate from mature adipose tissue and are categorized by their depth and location, such as subcutaneous, subfascial, intermuscular, and intramuscular [2–4]. Although their aetiology remains unclear, genetic, metabolic, and endocrine factors are believed to contribute to their development [4, 5]. Obesity is a significant factor, as obese individuals are more likely to develop lipomas than those maintaining a healthy weight [4]. Gene dysregulation resulting in decreased lipolysis and low cyclic adenosine monophosphate (cAMP) levels in adipocytes is thought to underlie lipid accumulation in lipomas. Superficial lipomas may model lipid buildup similar to that seen in obese individuals [8]. Intramuscular lipomas arise from the infiltration of mature adipocytes into muscle fibres, gradually exerting pressure on surrounding tissues and leading to clinical symptoms [9].

Clinically, lipomas present as soft, mobile, and painless masses. However, giant lipomas may cause symptoms such as nerve compression, lymphedema, and discomfort due to their size and location [4, 10]. In this case, the mass became noticeable following significant weight loss after bariatric surgery. Diagnostic imaging, including CT and MRI, is essential for evaluating the extent and nature of lipomas. While ultrasound (US) is a cost-effective, non-invasive initial imaging modality, CT and MRI provide more detailed assessments, particularly for deep-seated or large lipomas [5, 10].

Large intramuscular lipomas carry clinical implications, potentially causing functional limitations and nerve compression syndromes when located near critical structures [1]. Although imaging in this case indicated a benign lesion, surgical excision was performed to obtain a biopsy and exclude the possibility of malignancy.

Treatment options for lipomas include non-excisional techniques, enucleation, and surgical excision. Non-excisional treatments, such as steroid injections and liposuction, are becoming more common. Steroid injections, effective for small lipomas under 1 inch, induce local fat atrophy, reducing lesion size. Liposuction is advantageous for cosmetic reasons but can make complete removal challenging. Enucleation involves a small incision and curette to remove the tumour without requiring sutures.

For larger lipomas, surgical excision is the most reliable approach. It involves making an incision over the tumour, carefully dissecting it from surrounding tissues, and removing it entirely. However, excision carries potential complications, including surgical infections, hematomas, seromas, nerve and vascular injuries, cosmetic deformities, and fat embolism [11]. In this case, surgical excision was uneventful, though a postoperative seroma developed, which required drainage.

The prognosis for benign lipomas is excellent following complete excision, with low recurrence rates if the tumour is entirely removed [12]. However, managing patient expectations regarding cosmetic outcomes is crucial. Studies indicate that patients prioritize cosmetic results, with factors such as age, gender, and surgical site visibility influencing their satisfaction [13]. Preoperative counselling is essential to align expectations with realistic outcomes, as unrealistic expectations may reduce satisfaction despite objectively favourable results [14].

## Conclusion

This case underscores the importance of considering giant lipomas in the differential diagnosis of soft tissue masses in the scapular region. Accurate diagnosis requires thorough clinical examination and imaging, including US, CT, and MRI, to guide biopsy decisions and avoid unnecessary invasive procedures. Surgical excision with a recommended 1 cm margin and drain placement remains the preferred treatment to prevent local recurrence and complications such as hematomas or seromas. Postoperative pathological analysis is essential to confirm the diagnosis. While prognosis is generally favourable, recurrence remains possible.
